# Correction to “Tumor Necrosis Factor‐α Promotes the Lymphangiogenesis of Gallbladder Carcinoma Through Nuclear Factor‐κB‐Mediated Upregulation of Vascular Endothelial Growth Factor‐C”

**DOI:** 10.1111/cas.70127

**Published:** 2025-06-25

Qiang Du, Lei Jiang, Xiaoqian Wang, Meiping Wang, Feifei She, Yanling Chen. Tumor Necrosis Factor‐α Promotes the Lymphangiogenesis of Gallbladder Carcinoma Through Nuclear Factor‐κB‐Mediated Upregulation of Vascular Endothelial Growth Factor‐C. Cancer Science 105 (2014) 1261–1271. https://doi.org/10.1111/cas.12504.

In the above article, Figure 4d is incorrect. The correct image is shown below:

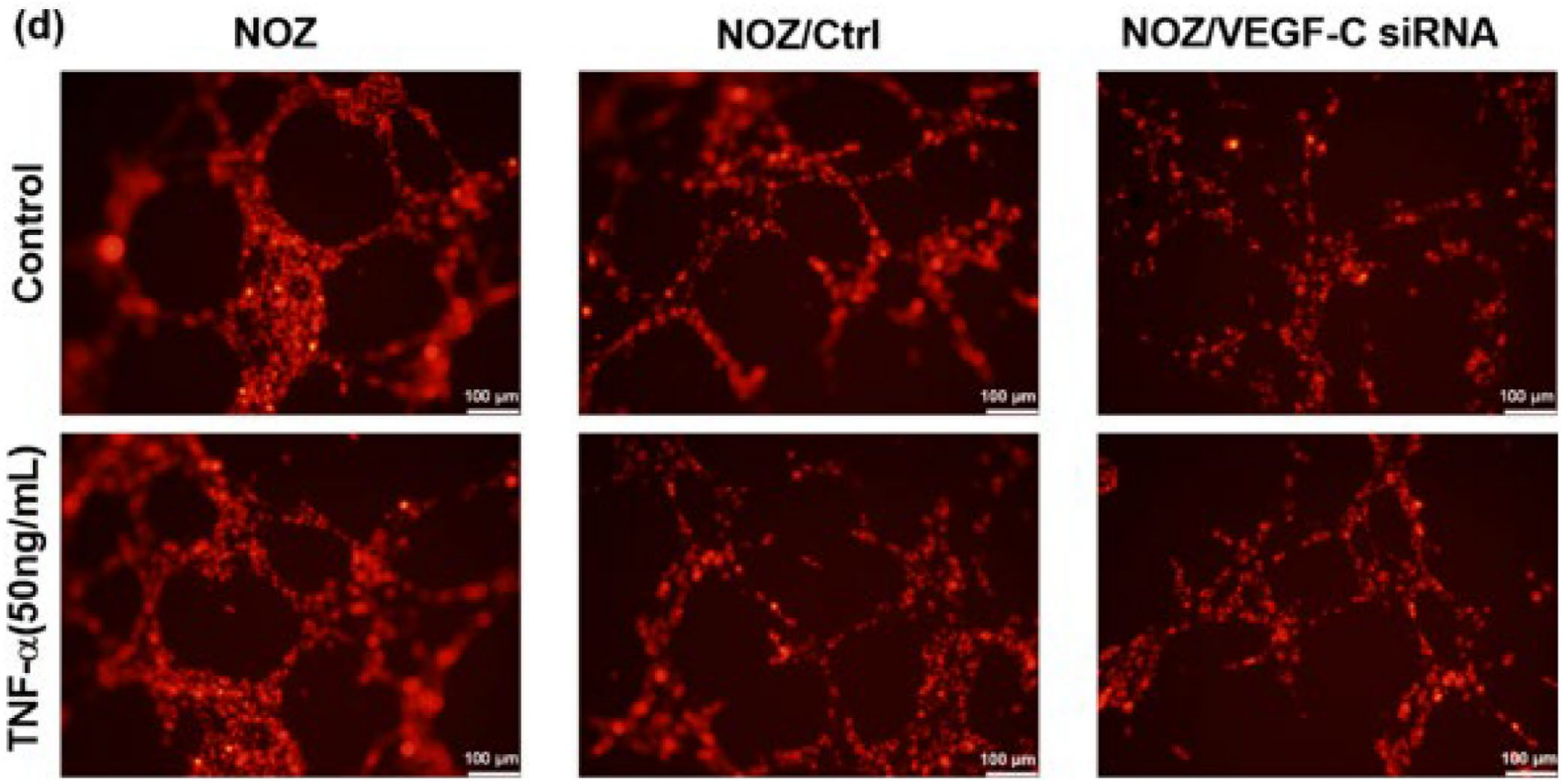

We apologize for this error.

